# Limitations of the MTB/RIF Assay: An Xpert Review of 4 Clinical Cases

**DOI:** 10.1093/ofid/ofaf132

**Published:** 2025-03-05

**Authors:** Allison L Haas, Angela Ma, Jonathan Pham, Punam Verma, Uma Malhotra, E Chandler Church, Masahiro Narita, Vincent Escuyer, Salika M Shakir

**Affiliations:** Department of Pathology, University of Utah School of Medicine, Salt Lake City, Utah, USA; Division of Infectious Diseases, ARUP Laboratories, Salt Lake City, Utah, USA; Department of Pathology, University of Utah School of Medicine, Salt Lake City, Utah, USA; Division of Infectious Diseases, ARUP Laboratories, Salt Lake City, Utah, USA; Department of Pathology, University of Utah School of Medicine, Salt Lake City, Utah, USA; Division of Infectious Diseases, ARUP Laboratories, Salt Lake City, Utah, USA; Department of Pathology and Laboratory Medicine, Virginia Mason Medical Center, Seattle, Washington, USA; Infectious Diseases, Virginia Mason Franciscan Health, Seattle, Washington, USA; Department of Medicine, Division of Allergy and Infectious Diseases, University of Washington, Seattle, Washington, USA; Department of Medicine, Division of Allergy and Infectious Diseases, University of Washington, Seattle, Washington, USA; Vaccine and Infectious Diseases Division, Fred Hutchinson Cancer Center, Seattle, Washington, USA; Seattle & King County Public Health Department, Seattle, Washington, USA; Seattle & King County Public Health Department, Seattle, Washington, USA; Department of Medicine, Division of Pulmonary, Critical Care and Sleep Medicine, University of Washington, Seattle, Washington, USA; New York State Department of Health, Wadsworth Center, Albany, New York, USA; Department of Pathology, University of Utah School of Medicine, Salt Lake City, Utah, USA; Division of Infectious Diseases, ARUP Laboratories, Salt Lake City, Utah, USA

**Keywords:** *Mycobacterium tuberculosis*, rifampin resistance, rpoB mutation, Xpert MTB/RIF, rapid diagnostics

## Abstract

Current U.S. Centers for Disease Control and Prevention tuberculosis (TB) guidelines recommend molecular testing for initial diagnosis of TB and detection of rifampin resistance to expedite initiation of proper treatment. The Cepheid Xpert MTB/RIF assay can detect members of the *Mycobacterium tuberculosis* complex and rifampin resistance by evaluating for mutations in the *rpoB* gene. However, false-positive and false-negative detection of *M tuberculosis* and rifampin resistance results can lead to incorrect treatment of patients, including overuse of second-line anti-TB drugs, and may result in patient harm and increased healthcare cost. We present a series of 4 cases to demonstrate the limitations of the Xpert MTB/RIF assay in the diagnosis of TB, emphasizing the importance of follow-up confirmatory testing and laboratory oversight in reporting accurate results.

Tuberculosis (TB), caused by the *Mycobacterium tuberculosis* (MTB) complex, infects an estimated 10.6 million people annually, resulting in approximately 1.3 million deaths [1]. Of these, it is estimated that approximately 3% of new cases and 18% of previously treated cases were multidrug- or rifampin-resistant in 2023, requiring treatment with second-line drugs [1]. To appropriately and promptly treat patients with active TB disease, particularly multidrug-resistant TB (MDR TB), and to mitigate spread of this public health threat, rapid diagnostic testing is essential because mycobacterial cultures and phenotypic susceptibility testing may take several weeks to yield results. The Cepheid Xpert *Mycobacterium tuberculosis* Complex (MTB) Detection and Rifampin Resistance by polymerase chain reaction (MTB/RIF PCR) is a cartridge-based nucleic acid amplification test that detects MTB complex DNA and putative rifampin resistance mutations in a conserved 81 base-pair (bp) region of the *rpoB* gene. Rifampin resistance is used as a surrogate for MDR-TB. The World Health Organization (WHO) issued policy recommendations for implementation of rapid molecular testing for pulmonary MTB via the Xpert assay as early as 2011. Several studies have evaluated the performance of the Xpert system on different off-label laboratory-validated extrapulmonary specimens to diagnose extrapulmonary TB. Currently, the WHO recommends the Xpert MTB/RIF and the Xpert Ultra (next-generation Xpert MTB/RIF, not available in the United States) as the initial diagnostic test on respiratory and extrapulmonary specimens [2] in patients suspected with pulmonary TB, HIV-associated TB, or with those with signs and symptoms of extrapulmonary MTB [3]. Despite the obvious advantages of rapid molecular testing, the Xpert MTB/RIF assay has notable limitations that necessitate confirmation with culture, phenotypic susceptibility testing, and sequencing [4, 5]. In this case report series, we highlight clinical scenarios resulting in false prediction of rifampin resistance and discuss the necessary steps to investigate discrepant molecular and phenotypic susceptibility testing.

## CASE 1

**Figure 1.**
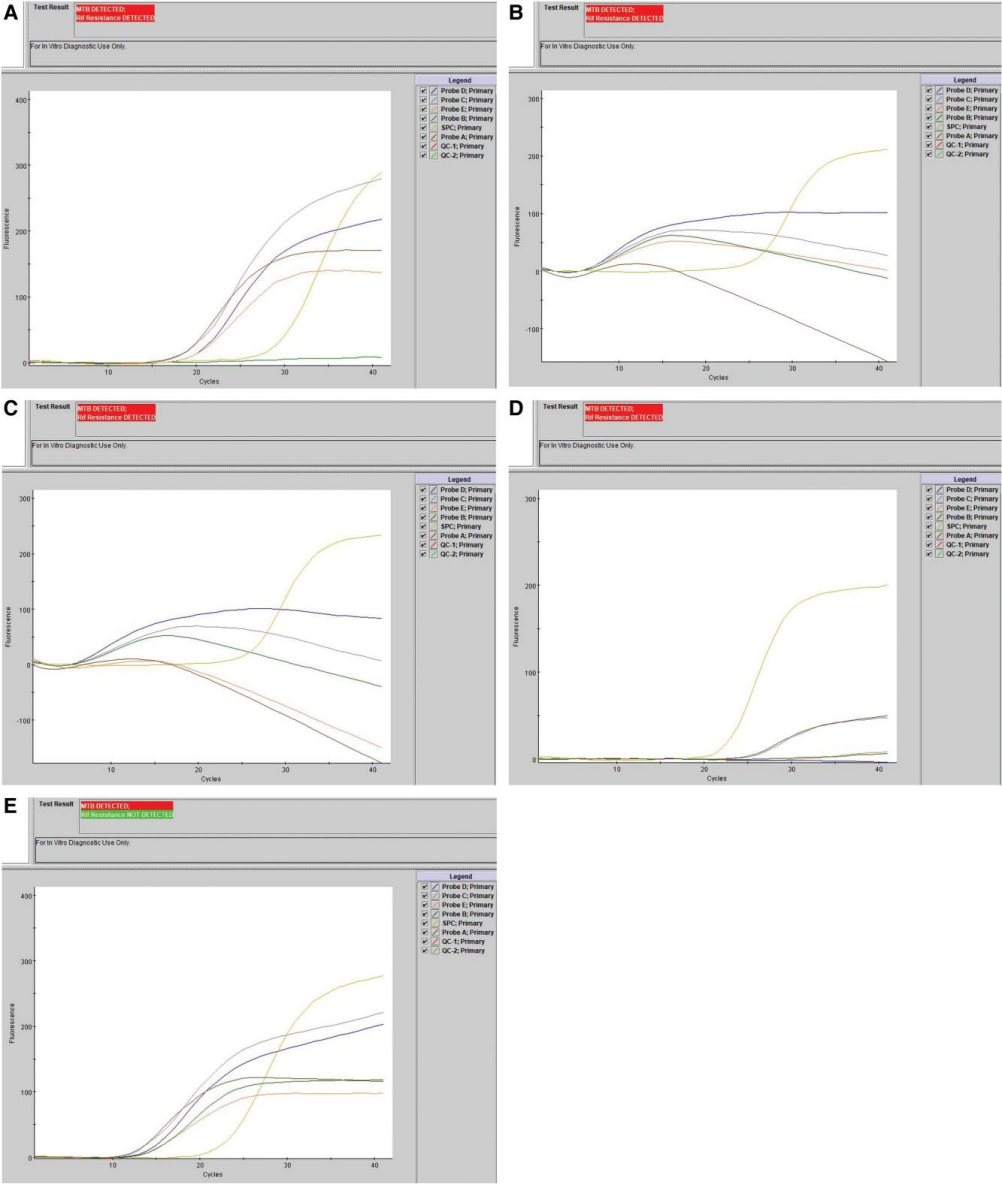
Xpert MTB/RIF PCR curves from (*A*) case 1, demonstrating false-positive detection of a silent mutation with no amplification in probe B. (b-c) Case 2, demonstrating false prediction of rifampin resistance due to high MTB bacterial load from sputum (*B*) and from pelvis cyst fluid (C). (*D*) Case 3, demonstrating false-positive detection of MTB and rifampin resistance due to cross-reaction with an atypical NTM. (*E*) Case 4, demonstrating a rifampin resistance-associated mutation present outside of the RRDR. Ct values of probes are as follows: a. case 1: A: 31.8, B: 33.6, C: 32.6. D: 33.9, E: 32.9, SPC: 29.7; b. case 2 (sputum): A: 0.0, B: 9.6, C: 9.8, D: 8.7, E: 0.0, SPC: 26.1; c. case 2 (pelvis cyst fluid): A: 0.0, B: 9.0, C: 9.1, D: 8.8, E: 10.0, SPC: 26.2; d. case 3: A: 24.4, B: 0.0, C: 25.3, D: 0.0, E: 0.0, SPC: 21.8; e. case 4: A: 14.2, B: 16.0, C: 14.5, D: 15.6, E: 16.5, SPC: 23.1. MTB/RIF PCR, *Mycobacterium tuberculosis* complex detection and rifampin resistance by polymerase chain reaction; Ct, cycle threshold; NTM, non-TB mycobacteria; RRDR, rifampin resistance determining region; SPC, specimen processing control.

A 38-year-old immunocompetent female presented to an urgent care clinic with night sweats, weight loss, dysuria, urinary urgency, and cloudy urine for approximately 2 months. She previously resided in Mexico and immigrated to the United States when she was 16 years old. She had no significant medical history or other comorbidities. A computed tomography (CT) scan of her abdomen and pelvis revealed severe left hydronephrosis, perinephric stranding, thickening of the ureter down to the mid-ureter, and peri-aortic lymphadenopathy. Purulent urine was surgically collected from the left kidney, which was sent for microbiological cultures. An auramine rhodamine (AO) fluorescent stain performed on the intraoperative urine specimens was acid fast bacilli (AFB) smear positive with 1+ AFB. Given the concern for renal TB, an Xpert MTB/RIF assay was performed on the concentrated urine, which was positive for the detection of MTB complex and rifampin resistance (Figure 1*A*). In the setting of MDR TB, the patient was started on a regimen including high-dose isoniazid, pyrazinamide, moxifloxacin, linezolid, and amoxicillin-clavulanic acid. The patient's urine sediment was sent to the Centers for Disease Control and Prevention (CDC)'s Molecular Detection of Drug Resistance (MDDR) service for DNA sequencing to evaluate for resistance-associated mutations.

The MDDR pyrosequencing and Sanger sequencing results revealed a silent mutation in the *rpoB* gene (TTC > TTT Phe431Phe [formerly Phe514Phe]) and an isoniazid resistance-associated mutation in the *inhA* promoter region (C-15T). No other resistance-associated mutations were identified. These results were confirmed by phenotypic drug susceptibility testing in the BD BACTEC MGIT system, which revealed resistance to a standard concentration of isoniazid (0.1 μg/mL), with susceptibility to rifampin (1.0 µg/mL), a high concentration of isoniazid (0.4 μg/mL), pyrazinamide (100 µg/mL), and ethambutol (5.0 µg/mL). After 10 weeks of treatment for MDR TB, the patient successfully completed a 9-month regimen of rifampin, high-dose isoniazid, pyrazinamide, and ethambutol following the results of the phenotypic susceptibility testing. Because of complications from renal tuberculosis, she developed autonephrectomy of the left kidney and required a left radical nephrectomy. AFB cultures from the removed kidney tissue were smear- and culture-negative for mycobacteria. The patient was monitored for a year following completion of therapy and surgery with no known recurrence of disease.

## CASE 2

A 75-year-old woman from India presented to her physician with low-grade fevers, tachycardia, leukocytosis, and progressive weakness for 2 weeks. She had a history of end-stage renal disease secondary to diabetic nephropathy and was on peritoneal dialysis before undergoing a living unrelated kidney transplant 6 months earlier. A CT scan of the abdomen and pelvis showed a 4.6-cm rim-enhancing septated complex cyst in the right adnexal region at the site of a previous peritoneal dialysis catheter. An AO stain performed on purulent drainage from the mass was smear positive with 4+ AFB with cording observed. Two days following drainage, she became critically ill with sepsis and respiratory failure requiring intubation. An AO stain on a sputum sample was also smear positive with 4+ AFB. She was started on a standard 4-drug regimen with isoniazid, rifampin, pyrazinamide, and ethambutol for possible TB.

An MTB/RIF PCR scan was performed on both the pelvis fluid and sputum and was positive for MTB complex and rifampin resistance (Figure 1*B*-*C*). The patient was initially transitioned to a bridging regimen of 5-drug therapy (high-dose pyrazinamide, amikacin, ethambutol, linezolid, and levofloxacin). Because of an increase in aspartate aminotransferase/alanine aminotransferase levels presumed to be related to pyrazinamide, the patient was transitioned to a WHO-recommended regimen for presumed MDR TB, consisting of bedaquiline, pretomanid, linezolid, and moxifloxacin (BPaLM). Mycobacterial cultures from the pelvis fluid, sputum, and blood grew MTB, confirming the diagnosis of miliary TB. However, phenotypic susceptibility testing revealed susceptibility to rifampin, isoniazid, ethambutol, and pyrazinamide. All isolates were sent to the CDC MDDR for DNA sequencing of resistance-associated mutations. Ultimately, no known mutations were identified to confer resistance to rifampin or any other antimicrobial, confirming that these isolates were pan-susceptible. Repeat MTB/RIF PCR performed on a 0.5 McFarland suspension of the cultured isolate in 7H9 broth from both the pelvis fluid and sputum resulted as positive for MTB complex, rifampin resistance not detected. During her 6 weeks of empiric MDR TB therapy, the patient experienced multiple medication toxicities including ocular toxicity secondary to linezolid, which in retrospect, was unnecessary. She completed a 9-month course of treatment including isoniazid and rifabutin and had a successful recovery. The patient is actively being monitored with no evidence of recurrence almost 1 year later.

## CASE 3

A 76-year-old male from the United States presented to the hospital with cough, shortness of breath, and respiratory failure requiring intubation. Upon evaluation, a chest radiograph identified a left upper lobe mass that was confirmed by a CT scan. His medical history included chronic obstructive pulmonary disease, hypertension, myasthenia gravis, and chronic kidney disease (stage 4). A transbronchial fine-needle aspiration sample was collected and sent for cytologic examination and a left upper lobe bronchoalveolar lavage (BAL) specimen was obtained for cytology and microbiologic cultures. An MTB/RIF PCR was also performed on the BAL specimen.

An AO stain on the BAL was negative, but the BAL MTB/RIF PCR detected MTB complex with predicted rifampin resistance (Figure 1*D*). The mycobacterial culture became positive after 19 days of incubation. Upon further workup and subculture to solid media, smooth yellow pigmented colonies were observed on Lowenstein-Jensen agar after 8 days of incubation, inconsistent with the colony morphology of MTB. Identification with matrix-assisted laser desorption ionization time of flight mass spectrometry was unsuccessful, and a 0.5 McFarland suspension in 7H9 broth from a pigmented colony was sent for partial 16S rRNA sequencing. Sequencing of the first 500 bp of the 16S rRNA gene did not provide a definitive species-level identification. Full-length 16S rRNA gene sequencing was performed and the sequence was compared against NCBI's *nt* database (including GenBank, EMBL, DDBJ, PDB, and RefSeq sequences), which provided an identification of *Mycolicibacterium moriokaense* with a low confidence score (98.5%) for species-level identification. Because of the pigmentation of the colonies on solid media and ability of the organism to grow within 8 days, this organism was reported as a rapidly growing pigmented mycobacteria, not MTB. No *M tuberculosis* was recovered in culture and no *rpoB* sequencing was performed. A repeat MTB/RIF PCR was performed directly from a 0.5 McFarland suspension of the cultured pigmented colonies and resulted as MTB complex detected, rifampin resistance predicted, confirming the results of the initial PCR. Upon further workup, the fine-needle aspiration was positive for adenocarcinoma with metastasis to the lymph nodes. The patient's clinical findings were ultimately attributed to cancer rather than infection. Antimicrobial treatment regimen is unknown as the patient was lost to follow-up for cancer treatment.

## CASE 4

A 48-year-old male from the United States presented with fever, cough, hemoptysis, and weight loss occurring over the course of several months. He had a complicated medical history and comorbidities including Lyme disease, liver cirrhosis, anemia, ascites, gout, alcoholism, and aspiration pneumonia, for which he had undergone multiple hospitalizations and procedures. He was diagnosed with cavitary pulmonary disease after multiple cavitary lung lesions were seen by chest X-ray and CT scan. Following imaging results, he had a positive QuantiFERON-TB test, suggestive of TB. To confirm this diagnosis, a bronchoscopy was performed, and bronchial wash specimens were sent for mycobacterial culture and MTB/RIF PCR.

His bronchial wash had 3+ AFB with cording seen on AO stain and MTB/RIF PCR was positive for MTB complex with rifampin resistance not predicted (Figure 1*E*). The mycobacterial culture became positive after 10 days of incubation, growing MTB. However, phenotypic susceptibility testing in the BD BACTEC MGIT system revealed mono-resistance to rifampin (1.0 μg/mL) and susceptibility to isoniazid, ethambutol, and pyrazinamide. To resolve the discrepancy between PCR and phenotypic susceptibility results, this isolate was sent to the Wadsworth Center at the New York State Department of Health for targeted Next-Generation Sequencing. Sequencing of *rpoB* revealed the presence of a Val170Phe mutation known to confer rifampin resistance, located outside of the 81-bp rifampin resistance-determining region (RRDR) targeted by the MTB/RIF PCR assay, confirming rifampin mono-resistant MTB. It is unknown how these results impacted patient treatment and outcomes.

## DISCUSSION

**Figure 2.**
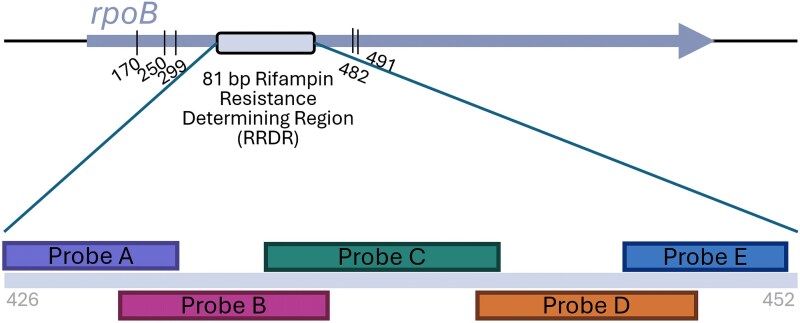
Xpert detection of rifampin resistance in *Mycobacterium tuberculosis rpoB*. The 5 probes utilized by the Xpert MTB/RIF PCR and their approximate binding location within the rifampin resistance determining region (RRDR) are illustrated (A-E) along with the known rifampin resistance-associated mutations present outside of the RRDR at the codons listed [15]. MTB/RIF PCR, *Mycobacterium tuberculosis* complex detection and rifampin resistance by polymerase chain reaction.

Use of the Cepheid Xpert MTB/RIF assay in conjunction with culture has been shown to yield clinical and cost benefits for the diagnosis of pulmonary TB, particularly in patients with HIV [6–8]. Sputum is currently the only specimen type with US Food and Drug Administration approval for use with the Xpert MTB/RIF assay [9]. However, laboratories may choose to internally validate additional specimen types such as cerebrospinal fluid, tissue, urine, and other body fluids for its use [10], as well as AFB smear-positive broth cultures [11]. The Xpert MTB/RIF is a single-use, cartridge-based nucleic acid amplification test that detects MTB complex DNA and putative rifampin resistance mutations to rapidly diagnose MDR TB. MDR TB is defined as MTB with resistance to at least rifampin and isoniazid. Because mono-resistance to rifampin without accompanying isoniazid resistance is still relatively rare, detection of rifampin resistance alone is often considered a surrogate marker for MDR TB. To detect potential rifampin resistance, an 81-bp RRDR of the MTB *rpoB* gene is amplified and evaluated for mutations, evidenced by loss of or decreased binding by at least 1 of 5 probes (probes A-E) (Figure 2). A total of 96% of known rifampin resistance mutations are found in the RRDR [12]. However, the Xpert MTB/RIF does not distinguish between silent and true mutations conferring rifampin resistance. Approximately 20% of mutations in the RRDR of the *rpoB* gene are silent (synonymous) mutations, which do not confer any amino acid substitutions leading to resistance [13]. Of these, the TTC > TTT Phe431Phe silent mutation detected by probe B of the Xpert MTB/RIF PCR assay, observed in case 1 (Figure 1*A*), is the most common and accounts for approximately 70% of false rifampin resistance [14]. However, silent mutations detected by probes A, D, and E, in addition to probe B, have also been associated with false prediction of rifampin resistance [5, 13].

Organism burden also plays an important role in detection and may result in both false-positive and false-negative results. Previous reports have described that low cycle threshold (Ct) values due to high bacterial load (>10^6^ CFU/mL) have resulted in false detection of MTB and predicted rifampin resistance [4]. To call a sample positive for MTB, at least 2 of the 5 probes must be detected. When 1 or more of the 5 probes are not detected or Ct values are delayed by >4 cycles, the assay also predicts rifampin resistance. It is well documented that excess amounts of nucleic acid result in reduced sensitivity and specificity of PCR-based assays, likely from the unequal dynamics of test components resulting in altered target hybridization and amplification [16]. High bacterial loads of *M marinum, M abscessus, M smegmatis, M pheli,* and *M aurum* have previously been associated with false-positive MTB detection [17]. Of these, high loads of *M abscessus* and *M smegmatis* have been called rifampin-resistant MTB due to nonspecific probe binding. In case 2, we describe an MTB-positive sample with a high bacterial load that was falsely called rifampin resistant, confirmed by phenotypic susceptibility testing and absence of *rpoB* gene mutations by CDC MDDR sequencing. This was likely also due to unequal hybridization dynamics, including competition for binding and a drop in exponential amplification of DNA due to excess target. This phenomenon can also be appreciated in the unusual nonsigmoidal shape of the amplification curves (Figures 1*B* and 1*C*). Upon reviewing this case, the test manufacturer suggested repeating the test using a diluted sample, but this could not be done as the original sample had been discarded. To further investigate this case, we performed repeat MTB/RIF PCR on the saved isolates from both the pelvis fluid and sputum. A 0.5 McFarland suspension in 7H9 broth from each isolate was tested and resulted as positive for MTB complex, but rifampin resistance not predicted upon repeat testing, likely because of the lower concentration of organisms in the reaction (Supplementary Figure 1A-B). Although we cannot rule out the existence of a minor subpopulation of rifampin-resistant MTB cells in the original sample that were lost during culture, the patient was treatment-naïve before initial presentation and did recover on a standard rifabutin-based therapy.

We also observed cross-reactivity with non-TB mycobacteria (NTM), most closely related to *M moriokaense*, as illustrated in case 3. It is possible that this organism shares a high *rpoB* sequence-level homology with the MTB *rpoB* gene, particularly in some of the primer and probe binding regions, which might account for erroneous amplification and detection by a few probes resulting in false-positive detection of MTB and prediction of rifampin resistance. The same results were also obtained when performing confirmatory testing on a single isolated yellow pigmented colony grown in 7H9 broth (0.5 McFarland suspension) (Supplementary Figure 1C), suggesting that this result was due to cross-reactivity rather than a mixed population of bacteria including MTB. In this case, only 2 of the 5 probes were detected with a delta Ct max of >4 that would be predicted for rifampin resistance. With this observation, the presence of an NTM may be considered in individuals from areas with low incidence of drug-resistant TB and initial negative smear results.

Despite the routine use of predicting rifampin resistance based solely off the RRDR, several mutations have been identified outside of the RRDR that confer rifampin resistance and may be falsely reported as rifampin susceptible by molecular methods, as observed in case 4. Mutations lying outside of the RRDR that may be missed by molecular methods include codons at locations 170, 250, 299, 482, and 491 (Figure 2) [18]. This is problematic because these patients would not be started on appropriate second-line therapy for treatment of MDR TB. Resolution of conflicting laboratory results and follow-up sequencing may also further delay initiation of appropriate therapy. Currently no other WHO-recommended molecular assays are able to detect these outside mutations. As such, confirmatory sequencing of *rpoB* remains the preferred method for detecting rifampin-resistance associated mutations. Any discrepancies among phenotypic drug susceptibility testing (DST) and molecular results should always be followed up with sequencing.

As recommended by the CDC and product manufacturer, phenotypic DST or confirmatory sequence-based testing is necessary for confirmation of MDR TB and initiation of appropriate patient therapy due to the limitation in the Xpert MTB/RIF's ability to distinguish silent and missense/nonsense mutations. In fact, the most recent American Thoracic Society/CDC/European Respiratory Society/Infectious Diseases Society of America guidelines for the treatment of drug-resistant TB recommend sequencing rifampin-resistant isolates for mutations conferring resistance to first-line agents, fluoroquinolones, and aminoglycosides [19]. The CDC MDDR service offers free targeted next-generation sequencing to evaluate for resistance-associated mutations for rifampin, isoniazid, pyrazinamide, ethambutol, fluoroquinolones, second-line injectables, bedaquiline, clofazimine, and linezolid [20]. Sequencing can be performed not only on MTB complex isolates, but also on nucleic acid amplification test-positive direct patient specimens from a variety of sources including respiratory, body fluids, cerebrospinal fluid, tissue, and urine. Sequencing of resistance-associated mutations also provides results earlier than phenotypic DST, which may take weeks or months. This offers clinicians the advantage of adjusting anti-TB therapy for patients while waiting for phenotypic DST results. As illustrated in case 2, DNA sequencing results may allow clinicians to stepdown from more toxic MDR TB therapy to the standard regimen of rifampicin, isoniazid, pyrazinamide, and ethambutol.

Guidelines recommend treatment of drug-sensitive TB with a 4- or 6-month course of defined antibiotic combinations [21]. In cases of MDR and XDR TB, tailoring individual patient therapy regimens is highly complex and typically requires consideration of patient comorbidities, contraindications, intolerance, and healthcare cost, even with newer regimens including bedaquiline, pretomanid, linezolid (BPaL) and BPaLM [19]. Furthermore, treatment duration may be significantly longer, and patients must be closely monitored for development of adverse effects and drug resistance. Given these complexities, potential toxicities, and need to promptly and accurately detect MDR TB, rapid molecular MTB/RIF results should be carefully considered when selecting therapies to minimize patient harm.

## CONCLUSIONS

Implementation of the Xpert MTB/RIF PCR assay revolutionized the diagnosis of MTB and MDR-TB and allowed for much faster initiation of effective anti-TB therapy. However, discrepancies between Xpert MTB/RIF PCR and phenotypic DST have highlighted the limitations of the Xpert MTB/RIF PCR assay including false-positive and false-negative detection of MTB and prediction of rifampin resistance (Table 1). Here, we highlight the impact of silent mutations, high bacterial loads, cross reactivity with NTM, and mutations occurring outside of the RRDR on the diagnosis of MTB and choice of anti-mycobacterial therapies. Detection of rifampin resistance by the Xpert MTB/RIF assay should always be interpreted with clinical context and patient epidemiology before switching treatment to second-line MDR TB regimens. Implementation of the second-generation Xpert Ultra assay may mitigate some of the issues described here. The Ultra assay features improved sensitivity with a slight decrease in specificity overall [22, 23] but an increased ability to differentiate silent mutations from true rifampin resistance mutations. However, it is not Food and Drug Administration–approved or commercially available in the United States.

**Table 1.** Considerations When Evaluating Xpert MTB/RIF Results for Rifampin Resistance

Xpert MTB/RIF Result	Reasons	Observation	Consideration
False-positive prediction of rifampin resistance	Silent mutation	No detection or delayed amplification of one or more probes	Determine which probe was not detected (majority of silent mutations observed in probe B) Confirm with *rpoB* gene sequencing
	High MTB load	Ct values <10 and nonsigmoidal shape of amplification curves (unequal PCR hybridization and inefficient probe binding)	Consider repeat test with dilution Confirm with phenotypic DST and *rpoB* gene sequencing
	Low or no bacterial load	Reduced amplification and reduced probe binding	Consider repeat testing Correlate with culture results
	Cross reactivity with non-tuberculous mycobacteria	Only 2 of 5 probes detected	Determine pretest probability Correlate with smear and culture results
False-negative prediction of rifampin resistance	Rifampin resistance mutations present outside the RRDR region	Typical sigmoidal curve with all probes detected	Correlate with phenotypic DST Confirm with *rpoB* gene sequencing

Abbreviations: DST, drug susceptibility testing; MTB, *Mycobacterium tuberculosis;* PCR, polymerase chain reaction; RRDR, rifampin resistance determining region.

Given the public health and patient care implications of TB diagnostics, the Xpert MTB/RIF assay is best suited to be performed by trained medical laboratory scientists in a full-service clinical microbiology laboratory rather than a STAT or point-of-care testing environment. Careful oversight and stewardship of MTB/RIF PCR results should be applied by the laboratory to prevent automatic or indiscriminate reporting of high-consequence results such as rifampin resistance, suggesting MDR TB. This is particularly relevant for U.S.-based clinical laboratories, as the prevalence of MDR TB in the United States was only 1.4% (100 cases) in 2023 [24]. Laboratories should consider review of any positive MTB/RIF PCR results, particularly those with rifampin resistance predicted, and consider factors including Ct values, curve shape, and probes detected (as discussed in Table 1), which may require procedural changes. Upon prediction of rifampin resistance, the laboratory or medical director should consult with the clinical care team to understand the relevant patient exposures and symptoms to determine the pretest probability of TB or MDR TB before releasing results. If not consulted, we encourage clinicians to contact their clinical laboratory to gather relevant information on the probes and Ct values to aid in interpretation. Careful considerations should be given to initial molecular results, particularly in patients with a new diagnosis of MDR-TB who have a low pretest probability or no known exposures to MDR-TB. In such cases, the Infectious Diseases Society of America recommends sequencing to confirm resistance-associated mutations [3, 25]. Considering the intricacies in diagnosis of active TB disease and its treatment, it is important to recognize that rapid molecular testing methods should always be interpreted with clinical context and confirmed with molecular sequencing, culture, and phenotypic DST.

## Supplementary Material

ofaf132_Supplementary_Data

## Supplementary Data

Supplementary materials are available at *Open Forum Infectious Diseases* online. Consisting of data provided by the authors to benefit the reader, the posted materials are not copyedited and are the sole responsibility of the authors, so questions or comments should be addressed to the corresponding author.

## Notes

***Author contributions.*** A. H., J. P., U. M., and P. V. wrote the case portion. A. H., A. M., and S. S. wrote the discussion. A. H., A. M., J. P., E. C. C., M. N., V. E, and S. S. edited the manuscript.

***Patient consent statement.*** No patient consent was obtained because all data were obtained retrospectively and covered under University of Utah IRB 24431.

***Financial support.*** No funding was used to support the writing of this case report.
