# Supplementary material for: Limitations of the MTB/RIF Assay: An Xpert Review of 4 Clinical Cases
Source: Open Forum Infect Dis. 2025 Mar 5;12(4):ofaf132. doi: 10.1093/ofid/ofaf132 (PMC11953001; doi:10.1093/ofid/ofaf132)
Supplement: ofaf132_Supplementary_Data [file ofaf132_supplementary_data.docx]

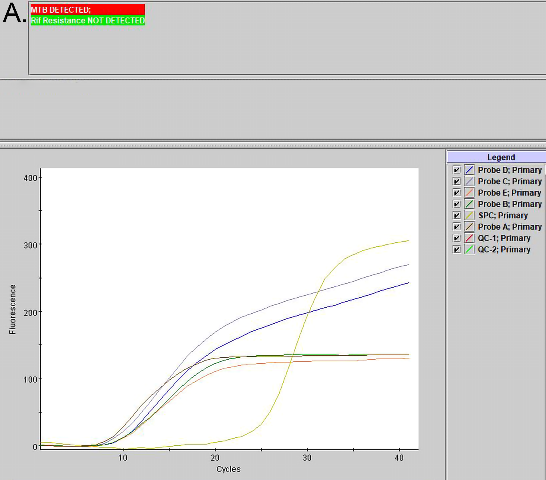

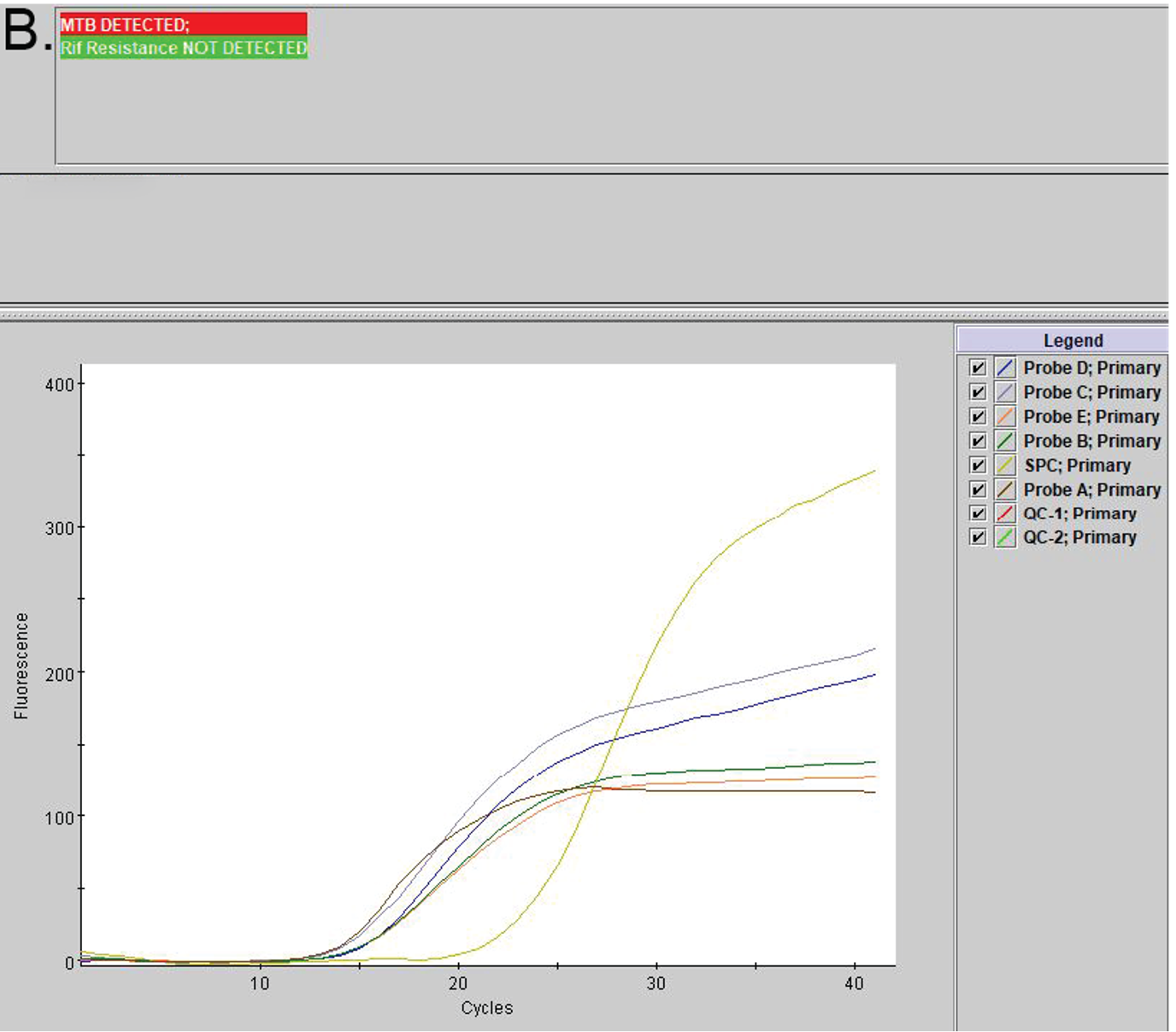

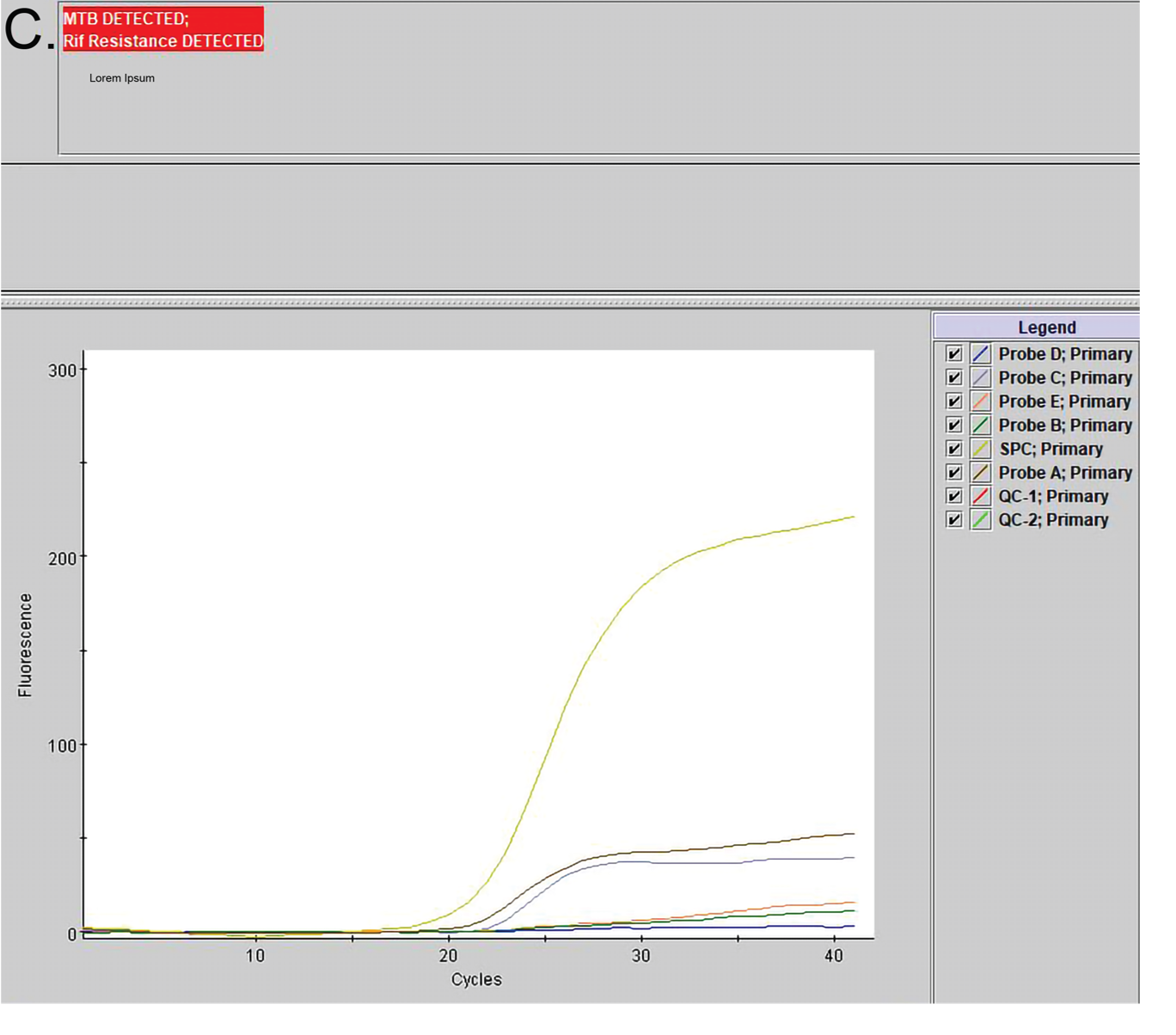


**Supplemental Figure 1. Repeat MTB/RIF PCR amplification curves for discrepant analysis.** A-B. Repeat amplification curves from pure 7H9 cultures (0.5 McFarland suspension) from Case 2, from sputum isolate (A) and pelvic cyst fluid isolate (B). C. Repeat amplification curves from Case 3 from single yellow isolated colony in 7H9 broth (0.5 McFarland suspension). CT values of probes are as follows: A. Case 2, sputum isolate: A: 9.8, B: 10.9, C: 10.3, D: 11.2, E: 11.5, SPC: 24.3. B. Case 2, pelvic cyst isolate: A: 15.3, B: 16.3, C: 15.6, D: 16.7, E: 16.9, SPC: 22.7. C. Case 3, yellow colony: A: 24.4, B: 0.0, C: 25.3, D: 0.0, E: 0.0, SPC: 21.8
